# Lectins at Interfaces—An Atomic Force Microscopy and Multi-Parameter-Surface Plasmon Resonance Study

**DOI:** 10.3390/ma11122348

**Published:** 2018-11-22

**Authors:** Katrin Niegelhell, Thomas Ganner, Harald Plank, Evelyn Jantscher-Krenn, Stefan Spirk

**Affiliations:** 1 Institute for Paper-, Pulp- and Fiber Technology, Graz University of Technology, Inffeldgasse 23, 8010 Graz, Austria; k.niegelhell@gmx.at; 2 Institute for Electron Microscopy and Nanoanalysis, Graz University of Technology, Steyrergasse 17, 8010 Graz, Austria; Thomas.ganner@a1.net (T.G.); harald.plank@felmi-zfe.at (H.P.); 3 Department of Obstetrics and Gynecology, Medical University of Graz, Auenbruggerplatz 14, 8036 Graz, Austria; evelyn.jantscher-krenn@medunigraz.at

**Keywords:** lectin, bovine serum albumin, adsorption, cellulose thin film, polystyrene, gold, surface plasmon resonance spectroscopy

## Abstract

Lectins are a diverse class of carbohydrate binding proteins with pivotal roles in cell communication and signaling in many (patho)physiologic processes in the human body, making them promising targets in drug development, for instance, in cancer or infectious diseases. Other applications of lectins employ their ability to recognize specific glycan epitopes in biosensors and glycan microarrays. While a lot of research has focused on lectin interaction with specific carbohydrates, the interaction potential of lectins with different types of surfaces has not been addressed extensively. Here, we screen the interaction of two specific plant lectins, Concanavalin A and Ulex Europaeus Agglutinin-I with different nanoscopic thin films. As a control, the same experiments were performed with Bovine Serum Albumin, a widely used marker for non-specific protein adsorption. In order to test the preferred type of interaction during adsorption, hydrophobic, hydrophilic and charged polymer films were explored, such as polystyrene, cellulose, *N*,*-N*,*-N*-trimethylchitosan chloride and gold, and characterized in terms of wettability, surface free energy, zeta potential and morphology. Atomic force microscopy images of surfaces after protein adsorption correlated very well with the observed mass of adsorbed protein. Surface plasmon resonance spectroscopy studies revealed low adsorbed amounts and slow kinetics for all of the investigated proteins for hydrophilic surfaces, making those resistant to non-specific interactions. As a consequence, they may serve as favorable supports for biosensors, since the use of blocking agents is not necessary.

## 1. Introduction

Lectins are a diverse group of carbohydrate binding proteins featuring at least one non-catalytic domain that reversibly binds to specific mono- or oligosaccharides [1]. These sugar binding proteins are commonly classified in terms of their source (i.e., plants, fungi, animals), or carbohydrate specificity (e.g., glucose/mannose, galactose, sialic acid or fucose) [2,3]. Lectins are critical for cell communication and signaling in many physiologic and pathophysiologic processes. The versatile structure of lectins results in a large diversity of properties, reaching from anti-insect, anti-tumor, immunomodulatory, antimicrobial to HIV-I reverse transcriptase inhibitor activities [4]. Many human pathogens (viral, bacterial or protozoan) employ lectins to bind to glycans displayed on the host’s cell surfaces and thus, initiate adhesion and infection. Escherichia coli, for instance, binds to mannosides and the Influenza virus attaches via sialic acid residues on the host’s cell surfaces. Aberrant cell surface glycosylation is a hallmark of tumor cells, and lectin interactions with tumor-specific glycan epitopes can promote tumor growth and immune modulation [5,6]. Therefore, lectins are subject to extensive studies in the fields of infectious diseases and cancer research as potential drug targets or therapeutic agents, as well as diagnostic and prognostic tools [2,7]. 

Other applications of lectins are their use in structural glycan analysis. The carbohydrate binding affinity of lectins is exploited for the detection of glycans or glycan containing molecules. Lectin microarrays are applied to separate, isolate and identify mono-, oligo- or polysaccharides, glycoproteins and glycolipids. Additionally, lectins are employed in biosensors to analyze lectin-carbohydrate interactions, such as specificity, affinity and kinetics [3]. When it comes to biosensors, protein adsorption is a very critical factor, since non-specific interactions of the protein with the substrate influence sensitivity and selectivity. Therefore, blocking agents are employed to minimize those factors [8]. 

Many parameters are affecting the adsorption behavior of proteins, among others the nature of the substrates, such as hydrophilicity or hydrophobicity and surface morphology. Therefore, fundamental adsorption studies assist to predict the behavior of proteins in the environment of a certain substrate e.g., used in a biosensor. Despite the countless number of lectin applications, there are only a few studies concerning non-specific adsorption of lectins. For instance, Amim et al. investigated the effect of the use of amino-terminated substrates for cellulose ester films and the concomitant change of surface free energy on the lectin-carbohydrate interaction [9] and Zemla et al. determined the preferred adsorption of lectins on parts of phase separated polymer thin films [10]. 

In this study, we examine the adsorption behavior of two lectins, Ulex Europaeus Agglutinin-I (UEA-I), a fucose binding lectin that is extracted from common gorse, and Concanavalin A (Con A), a lectin with mannose and glucose specificity extracted from jack bean [11,12]. Their adsorption behavior onto the different surfaces was compared to that of Bovine Serum Albumin (BSA), which is a widely used marker for non-specific protein interaction. The interaction capacity of the proteins with substrates of various kinds, such as hydrophilic, hydrophobic and charged (positively and negatively), was tested in real time by means of multi-parameter surface plasmon resonance spectroscopy (MP-SPR) in order to determine not only the adsorbed amount, but also the adsorption kinetics. The herein presented results give insight into the type of interaction that governs the adsorption behavior of these specific proteins.

## 2. Materials and Methods

**Materials.** Trimethylsilyl cellulose (TMSC, Avicel, M_w_ = 185,000 g·mol^−1^, M_n_ = 30,400 g·mol^−1^, PDI = 6.1 determined by GPC in chloroform) with a DS_Si_ value of 2.8 was purchased from TITK (Rudolstadt, Germany). Chloroform (99.3%), Toluene (99.9%), disodium phosphate heptahydrate (Na_2_HPO_4_ 7H_2_O), sodium dihydrogen phosphate monohydrate (NaH_2_PO_4_·H_2_O), hydrochloric acid (37%), sodium chloride (Ph.Eur.), sodium hydroxide (99%), polystyrene (PS, M_w_ = 35,000 g·mol^−1^), Bovine Serum Albumin (lyophilized powder, ≥96%, 66.5 kDa), Ulex Europaeus Agglutinin (lyophilized powder ≥80%, 63 kDa) and Concanavalin A (Type IV, lyophilized powder, 110 kDa) were purchased from Sigma Aldrich and used as received. *N*,*N*,*N*-trimethyl chitosan chloride (TMC, M_w_ = 90 kDa, medical grade, D_Acetylation_: 32%, DS_Me3+Cl_^−^: 66%) was purchased from Kitozyme, Belgium. Silicon wafers were cut into 1.5 × 1.5 cm^2^. SPR gold sensor slides (CEN102AU) were purchased from Cenibra, Bramsche, Germany. Milli-Q water (resistivity = 18.2 Ω^−1^·cm^−1^) from a Millipore water purification system (Millipore, Burlington, MA, USA) was used for contact angle and zeta-potential measurements and SPR investigations. 

**Figure A1:**
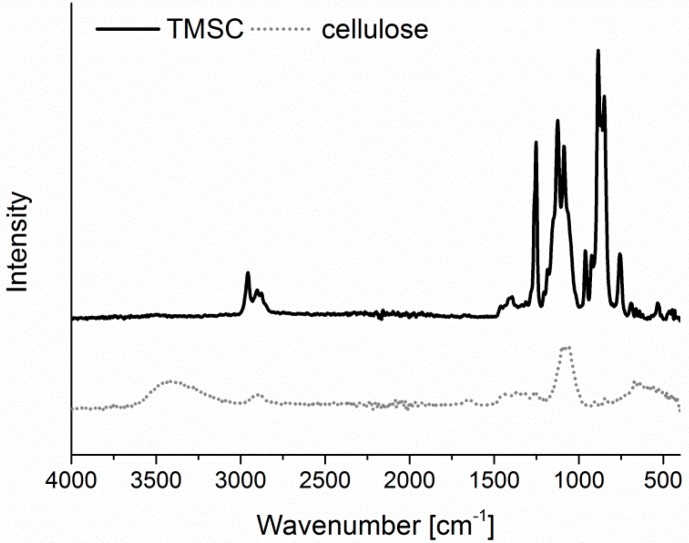
ATR-IR spectra of a TMSC thin film before and after regeneration. Conversion to cellulose upon HCl treatment is proven.

**Figure A2:**
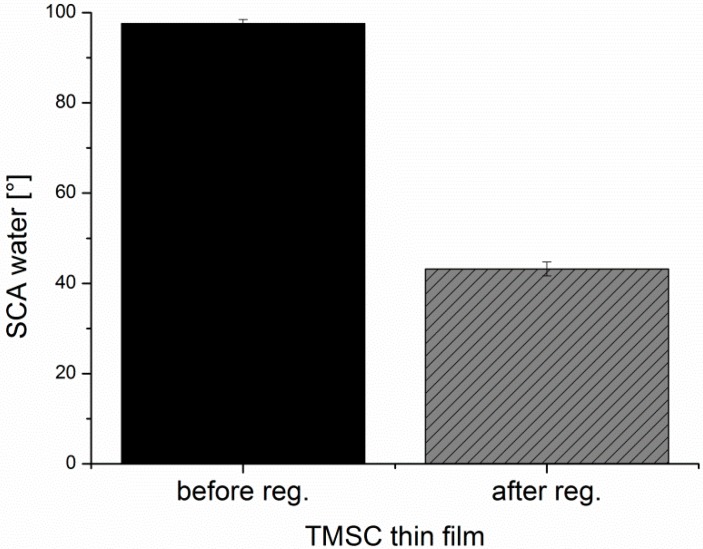
Static water contact angles of a TMSC thin film before and after regeneration to cellulose.

**Substrate Cleaning and Film Preparation.** Prior to spin coating, SPR gold sensor slides/silicon wafers were immersed in a “piranha” solution containing H_2_O_2_ (30 wt.%)/H_2_SO_4_ (1:3 *v*/*v*) for 10 min. Then substrates were extensively rinsed with Milli-Q water and blow dried with N_2_ gas. TMSC was dissolved in chloroform by stirring over night at room temperature and filtered through 0.45 μm PVDF filters. 120 µL of TMSC (1 wt.%) solution were deposited onto the substrate and then rotated for 60 s at a spinning speed of 4000 rpm and an acceleration of 2500 rpm·s^−1^. For converting TMSC into pure cellulose, the sensors/wafers were placed in a polystyrene petri-dish (5 cm in diameter) containing 3 mL of 10 wt.% hydrochloric acid (HCl). The dish was covered with its cap and the films were exposed to the vapors of HCl for 15 min. The regeneration of TMSC to cellulose was verified by ATR-IR (Figure A1) and water contact angle (Figure A2) measurements as reported elsewhere [13,14]. PS was dissolved in toluene by stirring over night at room temperature and filtered through 0.45 μm PVDF filters afterwards. 120 µL of PS (1 wt.%) solution were deposited onto the substrate and then rotated for 30 s at a spinning speed of 3000 rpm and an acceleration of 4500 rpm·s^−1^. TMC films were prepared by adsorption of TMC (1 mg·ml^−1^ dissolved in water, ionic strength was adjusted to 150 mM NaCl and pH value was adjusted to pH 7) onto cellulose substrates at a flow rate of 50 µL·min^−1^ for 5 min. TMC adsorption was monitored by MP-SPR.

**Infrared Spectroscopy.** IR spectra were attained by an Alpha FT-IR spectrometer (Bruker, Billerica, MA, USA) using an attenuated total reflection (ATR) attachment. Spectra were obtained in a scan range between 4000 to 400 cm^−1^ with 48 scans and a resolution of 4 cm^−1^. The data were analyzed with OPUS 4.0 software. 

**Profilometry.** Film thicknesses were acquired with a DETAK 150 Stylus Profiler from Veeco (Plainview, USA). The scan length was set to 1000 µm over a duration of 3 s. Measurements were performed with a force of 3 mg, a resolution of 0.333 µm per sample and a measurement range of 6.5 µm. A diamond stylus with a radius of 12.5 µm was used. Samples were measured after scratching the film (deposited on a silicon wafer). The resulting profile was used to calculate the thickness of different films. All measurements were performed three times.

**Contact Angle (CA) and Surface Free Energy (SFE) Determination.** Static contact angle measurements were performed with a Drop Shape Analysis System DSA100 (Krüss GmbH, Hamburg, Germany) with a T1E CCD video camera (25 fps) and the DSA1 v 1.90 software. Measurements were done with Milli-Q water and di-iodomethane using a droplet size of 3 µL and a dispense rate of 400 µL·min^−1^. All measurements were performed at least 3 times. CAs were calculated with the Young-Laplace equation and SFE was determined with the Owen-Wendt-Rabel-Kaelble (OWRK) method [15,16,17]. 

**Atomic Force Microscopy—AFM.** Surface characterization was done in ambient atmosphere at room temperature using two Multimode Quadrax MM and FastScanBio AFMs (both Bruker Nano, Billerica, MA, USA). While the former was operated with an NCH-VS1-W cantilever (NanoWorld AG, Neuchâtel, Switzerland, SUI) with force constants around 42 N·m^−1^, the latter used FastScan A cantilever (Bruker Nano, Billerica, MA, USA) with force constants around 18 N·m^−1^. Data analyses were done with the software packages Nanoscope (V7.30r1sr3, Veeco) and Gwyddion (V2.50). Image processing and in particular roughness analysis used line and/or plane fitting procedures together with cross-sectional analyses to remove curved and/or tilted background. No additional filters were used to prevent influence on data analyses. Root mean square (R_q_) values were derived from multiply selected area statistics to exclude unusually large particles (min. 3 images per sample were fully analyzed). Typical variation from area to area in the same, and in different images, vary less than 0.3 nm, which allows one to specify an accuracy range of ±0.2 nm for all Rq values.

**Zeta Potential Measurements.** The zeta potential measurements were performed by using a commercial electrokinetic analyzer (SurPASS™3, Anton Paar GmbH, Graz, Austria). For each sample, two zeta potential/pH value functions have been measured in 0.001 M KCl solution. For statistical reasons, four streaming potentials were measured at each pH value. The mean value of these data were used to calculate the potential/pH function.

**Multi Parameter Surface Plasmon Resonance Spectroscopy—MP-SPR.** MP-SPR spectroscopy was accomplished with an SPR Navi 210 from Bionavis Ltd., Tampere, Finland, equipped with two different lasers (670 and 785 nm, respectively) in both measurement channels, using gold coated glass slides as substrate (gold layer 50 nm, chromium adhesion layer 10 nm). All measurements were performed using a full angular scan (39–78°, scan speed: 8°·s^–1^). 

Gold sensor slides coated with the investigated thin films were mounted in the SPR, equilibrated with water and then with 10 mM PBS with an ionic strength of 100 mM NaCl at pH 5.5/7.4 (The pH value of the buffers was adjusted with 0.1 M hydrochloric acid or 0.1 M NaOH.). After equilibration, protein at a concentration of 0.1 mg·mL^−1^ (dissolved in the same buffer used for equilibration) is introduced into the flow cell. The protein is pumped through the cell with a flow rate of 50 µL·min^−1^ over a period of 5 min. After rinsing with buffer, the shift of SPR angle was determined and used to evaluate the amount of adsorbed protein. After protein adsorption all samples were rinsed with Milli-Q water and dried in a stream of N_2_ gas. All experiments have been performed in three parallels.

Protein adsorption was quantified according to Equation (1), which considers the dependence of the angular response of the surface plasmon resonance in dependence of the refractive index increment (*dn*/*dc*) of the adsorbing layer [18].

(1) $$\Gamma =\frac{\Delta \Theta \times k\times d_{p}}{dn/dc}$$

For thin layers (<100 nm), *k* × *d_p_* can be considered constant and can be obtained by calibration of the instrument by determination of the decay wavelength l_d_. For the SPR Navi 210 used in this study, *k* × *d_p_* values are approximately 1.09 × 10^–7^ cm/° (at 670 nm) and 1.9 × 10^–7^ cm/° (at 785 nm) in aqueous systems. For proteins, *dn*/*dc* in water-based buffer systems was reported 0.187 cm^3^·g^–1^, which was used to calculate the amount of adsorbed masses [19]. For TMC, the *dn*/*dc* value of chitosan (0.192 cm^3^·g^–1^) [20] was used for the calculation of adsorbed mass.

## 3. Results and Discussion

**Figure 1:**
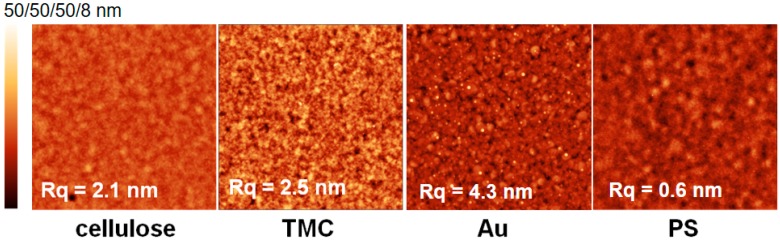
Atomic force microscopy height images (3 × 3 μm^2^) of the different substrates and corresponding RMS roughness (R_q_). All images are 3 × 3 µm^2^ while Z scales are 50 nm for cellulose, *N*,*N*,*N*-trimethyl chitosan chloride (TMC) and Au and 8 nm for polystyrene (PS) to visualize surface features.

In order to provide a variety of interaction possibilities for the proteins, hydrophobic polystyrene (PS), gold and hydrophilic substrates, such as negatively charged cellulose and positively charged *N*,-*N*,-*N*-trimethyl chitosan (TMC) were chosen as substrates for this adsorption study. Prior to adsorption experiments, the materials were characterized in terms of film thickness, surface free energy and morphology. As gold substrates, cleaned SPR sensor slides consisting of 50 nm gold deposited on a glass substrate with an adhesion layer of chromium in between (as reported from manufacturer), were used. Cellulosic substrates were prepared from spin coating trimethylsilyl cellulose (TMSC, Hsinchu City, Taiwan) and subsequent regeneration to cellulose by treatment with HCl vapors, which yielded thin films with a thickness of 30 ± 2 nm as determined by stylus profilometry measurements. Polystyrene films were spin coated as well, leading to film thicknesses of 58 ± 1 nm. The thickness of the positively charged TMC substrate could not be determined, since the substrate was prepared by adsorption of TMC onto cellulose resulting in thicknesses that were too low for detection with stylus profilometry. The different substrates were then subjected to atomic force microscopy (Figure 1). The high root-mean-square-RMS roughness (R_q_ = 4.3 nm) of the gold substrate is caused by the cleaning procedure with piranha, a very harsh treatment that removes all of the adventitious carbon that was adsorbed from the atmosphere [21]. The cellulosic and TMC substrate display similar RMS roughness (R_q_ = ca. 2 nm) originating from homogeneous TMC adsorption, thereby forming a thin layer on the cellulose film. The PS thin films show the lowest RMS roughness (R_q_ = 0.6 nm). All of the substrates are very homogenous and free of any visible contamination or pin-holes. 

**Figure 2:**
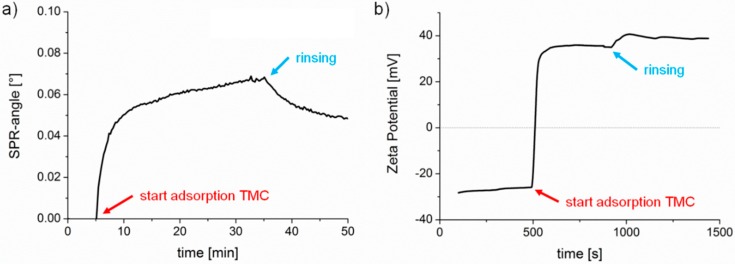
Adsorption of TMC on cellulose thin films. (**a**) Multi-parameter surface plasmon resonance spectroscopy (MP-SPR) sensogram measured at 785 nm, (**b**) zeta potential measurements.

As mentioned above, the positively charged substrate was prepared by adsorption of TMC onto cellulose thin films. Modification of cellulose substrates with TMC as an approach to control nonspecific protein adsorption behavior (using BSA) was already reported earlier [22,23] and the appropriate adsorption conditions for preparation of the cationic TMC substrates were adopted from these studies. In this work, TMC adsorption was monitored by multi-parameter surface plasmon resonance spectroscopy (MP-SPR) and zeta potential measurements (Figure 2). First, we observed a steady equilibration (rinsing with buffer) signal with MP-SPR, associated with a negative zeta potential (ca. −27 mV) for the pure cellulose film. Upon injection of the TMC solution, the SPR-angle increased and the zeta potential changed to positive values indicating deposition of TMC on the surface. Loosely bound material was clearly removed upon rinsing. However, the zeta potential of the adsorbed TMC layer shifted when rinsed with buffer to higher values (from 35 mV to 38 mV), which might be due to a change in conformation of the adsorbed layer. After adsorption, the MP-SPR sensogram showed a change of SPR-angle of 0.05°, which corresponds to an adsorbed amount of 0.6 ± 0.05 mg·m^−2^. For comparison, cationic starches with a similar charge density as TMC adsorbed to a much higher extent (1.2 mg·m^−2^) as shown recently [24]. 

Compared to the other substrates used in this study, TMC is the only one featuring a positive zeta potential. According to literature, the employed and cleaned gold surface displays a negative zeta potential above pH 5 [25], and PS also exhibits negative surface charge (−20 to −30 mV close to pH 7) [26]. The negative zeta potential for the cellulose thin films used in this study is supported by values reported in the literature on cellulosic fibers (−13 mV to −17 mV at pH 4.7–7.2) [22]. 

**Figure A3:**
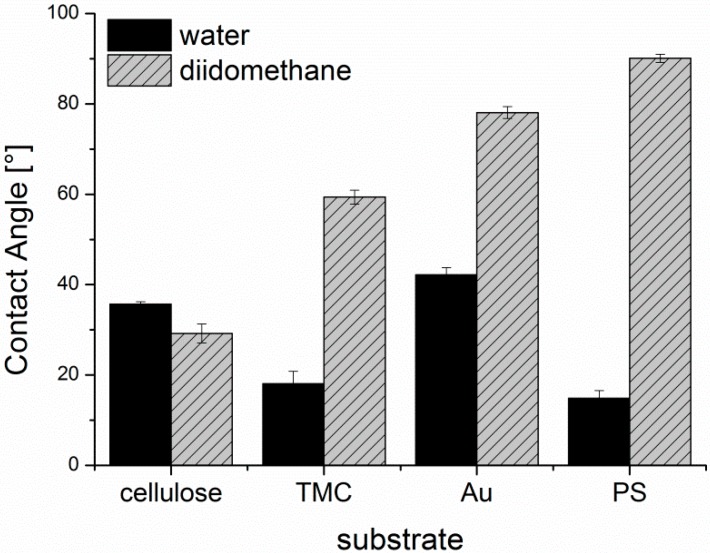
Contact angles measured with water and diiodomethane of different substrates used in the adsorption study. This data were used to calculate the surface free energies of the substrates by the OWRK method.

**Figure A4:**
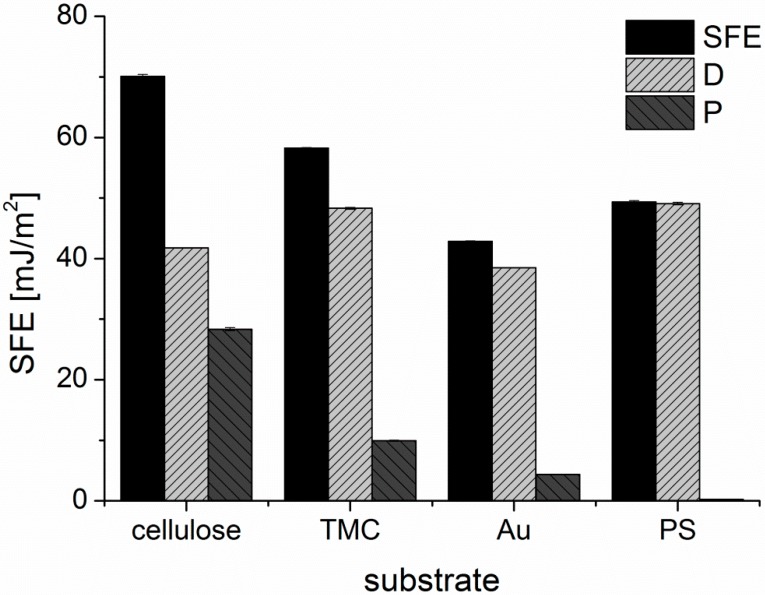
Surface free energies (SFE) and disperse (D) and polar (P) contributions to the SFE of different substrates used in the adsorption study.

The surface free energies (SFE) of the substrates were calculated from static contact angle measurements (Figure A3) and are presented in Figure A4. Cellulose and TMC surfaces both display a hydrophilic character. Although TMC displays a higher zeta potential than cellulose, cellulose shows higher SFE and larger polar contributions than TMC, which could be attributed to the different conformation of the adsorbed polymer in the dry state during contact angle measurements compared to the wet state in the zeta potential determination. PS exhibits, as expected, a hydrophobic surface without any significant polar contribution to the SFE. The lowest SFE of the investigated substrates is presented by the gold substrate. It is important to note, that the gold substrates were immediately used after the cleaning procedure for the adsorption experiments and for the other characterization tests. Thereby, it is guaranteed that the determined SFE is representative of all the samples in this work. 

**Figure 3:**
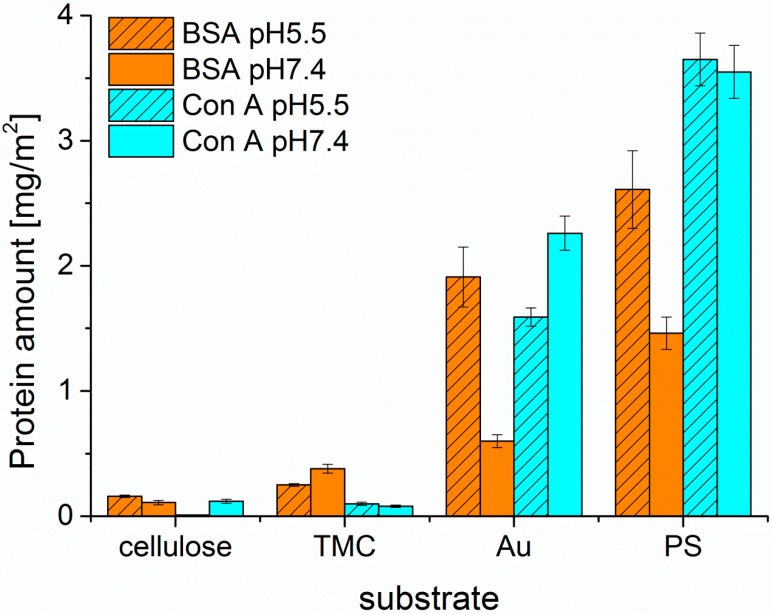
Comparison of adsorbed amount of Bovine Serum Albumin (BSA) and ConA calculated from the change in SPR-angle for different substrates at two pH values.

**Figure 4:**
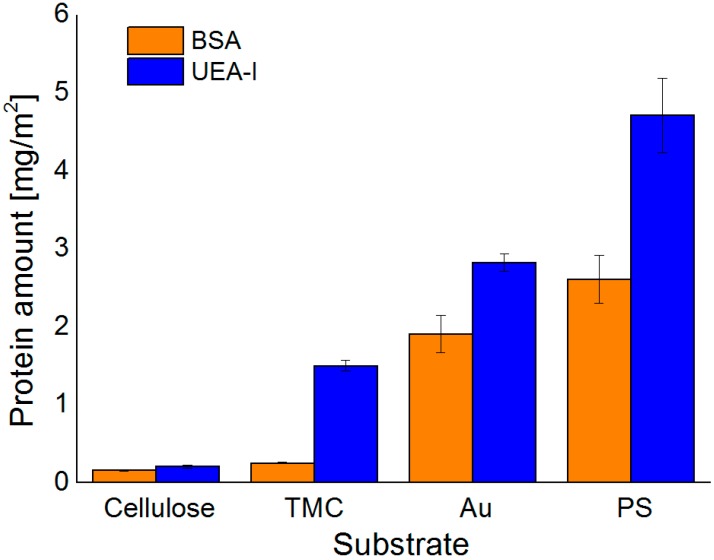
Comparison of adsorbed amount of BSA and UEA-I calculated from the change in SPR-angle for different substrates at pH 5.5.

After proper characterization of the substrates, the adsorption behavior of the different proteins was monitored by MP-SPR and the adsorbed amounts were calculated by the change in SPR-angle (Figure 3 and Figure 4). All of the examined proteins did adsorb to the least extent on the cellulose surface and to the highest on PS. This can be attributed to the apolar nature of the PS substrate leading to hydrophobic effects between protein and substrate. In general, hydrophobic effects in proteins are very common and are influencing the folding of proteins in aqueous environments. In such environments, the hydrophobic moieties are buried inside the protein minimizing their free energy. At hydrophobic surfaces, rearrangements of the proteins can take place by exposing the hydrophobic moieties towards that surface. This may even lead to denaturation of the protein, if the degree of interaction is very high. It is widely accepted that this process is mainly governed by entropic contributions rather than enthalpy, unless specific interactions come into play [27]. However, it should be noted here, that also non-hydrophobic effects may contribute to entropy, such as changes in low energy vibrational states.

There is less electrostatic repulsion between surface and protein in the case of PS than for cellulose, still the hydrophobic effect overrules the electrostatic attractions as seen by comparison of PS and TMC. At the investigated pH values, all of the proteins are negatively charged, because the pH values are above the isoelectric points (IEP_BSA_ = pH 4.7, IEP_Con A_ = pH 4.5–5.5, IEP_UEA-I_ = pH 4.8) [28,29]. Enhanced protein adsorption was observed when adsorbing proteins onto TMC modified cellulose compared to pure cellulose. Since TMC is positively charged, more electrostatic attraction takes place, whereas in the case of negatively charged cellulose, proteins are rather compelled at the investigated pH values [30]. The low protein adsorption on cellulose surfaces can be further rationalized by their high water content (ca 60 wt.%). Proteins are highly hydrated molecules as well and any removal of water will lead to a reduction in entropy. However, upon protein adsorption, water needs to be removed from the protein in order to irreversibly adsorb on the surface. Since this is, as we stated above, entropically unfavorable, cellulose surfaces (as well as nearly all highly swollen surfaces) are rather resistant towards non-specific protein deposition and fouling [31].

In general, the highest extent of protein adsorption is reached at the pH value near the isoelectric point, where the proteins exhibit a zero net charge. The balance of positive and negative charges leads to reduced solubility at pH 5.5 for all three proteins investigated, whereas at pH 7.4 the proteins are negatively charged, which increases solubility and causes smaller adsorbed amounts onto the surfaces. This effect is highly pronounced for BSA on all the examined surfaces, except for the TMC substrate. Since TMC is positively charged, it prefers the interaction with the negatively charged BSA at pH 7.4 rather than the more or less neutral BSA at pH 5.5. As for Con A, adsorption onto hydrophilic substrates was extremely low; for cellulose at pH 5.5 it was not even detectable. UEA-I was only investigated at pH 5.5 and showed the highest interaction capacity of all proteins and all pH values with all types of surfaces (Figure 4). Another factor affecting solubility, and subsequently protein deposition, is the aggregation of the proteins in solution, which may take place upon a change in pH value. For ConA, the dimers present at a pH of 5.5 are transformed into tetramers in solution at a pH value larger than 6 [32]. As a consequence, the solubility at the interface is reduced leading to larger deposited amounts in the case of non-specific interaction, which is indeed the observation for the Au and—to some extent—for the PS surfaces. For the latter, the additional complication is the rather large amount of deposited ConA, corresponding to a multilayer. It is known that upon the growth of such protein multilayers, at a certain point the surface reaches saturation and no more protein is adsorbed beyond this limit. 

**Figure 5:**
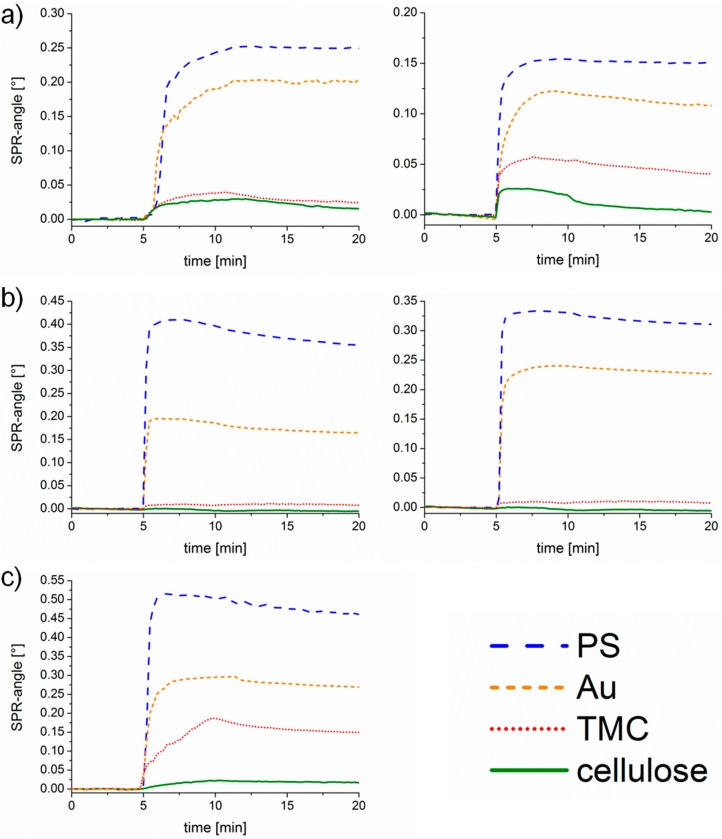
Sensograms measured by MP-SPR at 785 nm for different proteins; (**a**) BSA, (**b**) Con A and (**c**) Ulex Europaeus Agglutinin-I (UEA-I) at a pH value of 5.5 (**left**) and a pH value of 7.4 (**right**).

The sensograms, as shown in Figure 5, give an insight into the adsorption behavior observed in real-time and thereby allow for making statements on the kinetics. BSA adsorbs extremely fast (steep slope and quickly reaching an equilibrium) at pH 7.4 onto PS and cellulose, whereas adsorption is rather slow at pH 5.5. The interactions with PS and gold are strong since no material is removed upon rinsing. Only minor adsorption of Con A is detectable on the cellulose and TMC surface. However, extremely fast adsorption onto PS and gold is monitored indicating a high affinity to the substrates. At pH 7.4 only small amounts detached during rinsing, whereas at pH 5.5 an overshoot effect occurs, which is in the case of proteins usually explained by the so-called rollover model describing a reorientation of end-on into side-on adsorbed proteins [33]. The same effect is observed for UEA-I adsorption onto PS. Adsorption of UEA-I onto TMC and cellulose is very slow, not even reaching an equilibrium in the observed timeframe.

**Figure 6:**
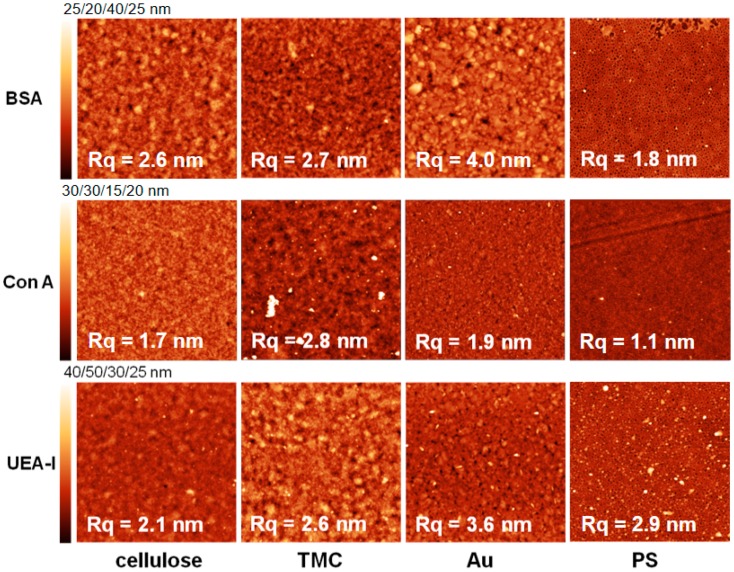
Atomic force microscopy height images of different substrates after protein adsorption at pH 5.5. All images are 3 × 3 µm^2^ while Z scales were individually adapted as specified above the scale bars in each row.

**Figure 7:**
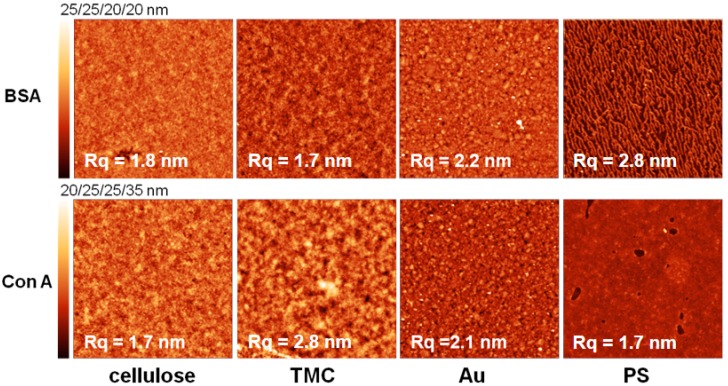
Atomic force microscopy height images of different substrates after protein adsorption at pH 7.4. All images are 3 × 3 µm^2^ while Z scales were individually adapted as specified above the scale bars in each row.

All of the surfaces were rinsed with water, dried and measured with atomic force microscopy (AFM) directly after protein adsorption. The images (Figure 6 and Figure 7) depict the adsorbed amount obtained by MP-SPR. For some surfaces there is hardly any change in surface topography, because the adsorbed amount was too low to be detected by AFM. In general, the more protein adsorbed on the surfaces, the lower the roughness of the surfaces was. This is an indication for preferable adsorption into valleys/pores of the substrates. However, for the extremely flat surface of PS (R_q_ = 0.6 nm), it is vice versa meaning that the roughness increases upon protein deposition. There, the proteins form island like features that fuse into a patch like morphology with increasing adsorbed amount before full coverage is achieved [34]. This is represented best by comparing the AFM images of BSA adsorbed onto PS. At pH 5.5 we observe nearly full coverage with a roughness of 1.8 nm, whereas at pH 7.4 islands of BSA lead to a higher roughness (2.8 nm).

## 4. Conclusions

The results of this adsorption study can be rationalized in the following way. The adsorption behavior of the examined lectins is comparable to BSA in terms of affinity to substrates of different types. The largest adsorbed amounts and fastest kinetics were observed on the PS surface indicating that hydrophobic effects govern the attraction of the investigated proteins to the substrate, which in turn are mostly driven by entropic contributions. The preferred adsorption onto gold is most likely enhanced by interactions of the thiol groups of the proteins (e.g., methionin for ConA), because of the good interaction capacity of sulfur and gold. The affinity to the hydrophilic substrates was exceptionally low, even when positive charges were introduced by adsorbing TMC. In addition, both types of polysaccharide layers are very prone to swelling (water contents up to 60 wt.%), which impedes protein adsoprtion via entropy since some of the water must be removed from the system in order to accomplish for protein deposition. Although Con A is a mannose/glucose binding lectin, which could interact with the glucose residues from cellulose, no adsorption was detected, probably due to the small number of available end groups of the cellulose. 

In conclusion, the binding interactions of BSA, UEA-I and Con A are primarily based on hydrophobic effects, therefore hydrophilic substrates, such as cellulose and TMC, compared to for instance PS, offer huge advantages for the utilization in biosensor development. They are not only stemming from renewable resources, but when used as a support material they are resistant to non-specific protein adsorption thereby avoiding the introduction of blocking agents. As a consequence, they are highly suitable to be used for a variety of lectin-based arrays. Future research will focus on interactions of human milk oligosaccharide with specific lectins immobilized on polysaccharide surfaces, which is an important topic concerning the health of breastfed new-born children.
